# Non-additive effects of electrical stimulation of the dorsolateral prefrontal cortex and the vestibular system on muscle sympathetic nerve activity in humans

**DOI:** 10.1007/s00221-024-06852-5

**Published:** 2024-06-01

**Authors:** Brendan McCarthy, Sudipta Datta, Gianni Sesa-Ashton, Rebecca Wong, Luke A Henderson, Tye Dawood, Vaughan G Macefield

**Affiliations:** 1 Baker Heart and Diabetes Institute, Melbourne, VIC Australia; 2 Baker Department of Cardiometabolic Health, The University of Melbourne, Melbourne, VIC Australia; 3 School of Medical Sciences (Neuroscience), Brain and Mind Centre, The University of Sydney, Camperdown, NSW Australia; 4 Department of Neuroscience, School of Translational Medicine, Monash University, The Alfred Centre, 99 Commercial Road, Melbourne, VIC 3004 Australia

**Keywords:** Dorsolateral prefrontal cortex, Muscle sympathetic nerve activity, Rostral ventrolateral medulla, Transcranial electrical stimulation, Vestibular system

## Abstract

Sinusoidal galvanic vestibular stimulation (sGVS) induces robust modulation of muscle sympathetic nerve activity (MSNA) alongside perceptions of side-to-side movement, sometimes with an accompanying feeling of nausea. We recently showed that transcranial alternating current stimulation (tACS) of the dorsolateral prefrontal cortex (dlPFC) also modulates MSNA, but does not generate any perceptions. Here, we tested the hypothesis that when the two stimuli are given concurrently, the modulation of MSNA would be additive. MSNA was recorded from 11 awake participants via a tungsten microelectrode inserted percutaneously into the right common peroneal nerve at the fibular head. Sinusoidal stimuli (± 2 mA, 0.08 Hz, 100 cycles) were applied in randomised order as follows: (i) tACS of the dlPFC at electroencephalogram (EEG) site F4 and referenced to the nasion; (ii) bilateral sGVS applied to the vestibular apparatuses via the mastoid processes; and (iii) tACS and sGVS together. Previously obtained data from 12 participants supplemented the data for stimulation protocols (i) and (ii). Cross-correlation analysis revealed that each stimulation protocol caused significant modulation of MSNA (modulation index (paired data): 35.2 ± 19.4% for sGVS; 27.8 ± 15.2% for tACS), but there were no additive effects when tACS and sGVS were delivered concurrently (32.1 ± 18.5%). This implies that the vestibulosympathetic reflexes are attenuated with concurrent dlPFC stimulation. These results suggest that the dlPFC is capable of blocking the processing of vestibular inputs through the brainstem and, hence, the generation of vestibulosympathetic reflexes.

## Introduction

It has long been established that gravity is capable of influencing the distribution of blood throughout the body. When habitually upright humans change from a prone or supine position to an upright one, blood begins to pool towards the feet (Olufsen et al. 2005; Yates et al. 2014). The cardiopulmonary and arterial baroreceptors work through the baroreflex to offset this tendency, yet they are known to be relatively slow to activate (Sagawa 2011; Wieling et al. 2007). As such, the vestibular system acts in a feed-forward manner via the sympathetic nervous system to aid the baroreflex with rapid adjustments to blood pressure (BP) through muscle sympathetic nerve activity (MSNA) (Holstein et al. 2011; McCarthy et al. 2023b; Voustianiouk et al. 2006). MSNA itself is comprised of bursts of vasoconstrictor impulses linked to the cardiac cycle via the arterial baroreflex (Fatouleh et al. 2013; Hammam and Macefield 2017) and can be recorded via percutaneous insertion of a microelectrode into a peripheral nerve (microneurography) (Macefield 2013; Vallbo et al. 2004). A proven method of selectively activating the vestibular system to induce these changes in sympathetic outflow is the use of sinusoidal galvanic vestibular stimulation (sGVS), which has been shown at low-frequencies to elicit both primary and secondary peaks of MSNA (Bent et al. 2006; Cohen et al. 2012; Hammam et al. 2011). The sinusoidal, binaural current induces perceptions of side-to-side movement in participants, similar to that of ‘rocking in a boat’ or ‘swaying in a hammock’.

In order to modulate changes in MSNA, vestibular signals are sent through a relay system in the medulla oblongata alongside the circuitry responsible for the baroreceptor reflex. Arterial baroreceptor afferents project to the caudal part of the nucleus of the solitary tract (NTS), which in turn sends excitatory information to the caudal ventrolateral medulla (CVLM) (Colombari et al. 2001; Holstein et al. 2011). Neurones in the CVLM exert inhibitory control of the rostral ventrolateral medulla (RVLM), which is the primary nucleus containing premotor vasoconstrictor neurones supplying skeletal muscle (Dampney 1994). Importantly, the dorsolateral prefrontal cortex (dlPFC) has also been implicated in the regulation of the sympathetic nervous system. Spontaneous bursts of MSNA at rest positively correlated with bilateral activity in the dlPFC, as James et al. (2013) assessed via concurrent recordings of MSNA and functional magnetic resonance imaging (fMRI) of the brain: bursts of MSNA were temporally coupled to an increase in BOLD (Blood-Oxygen-Level-Dependent) signal intensity in the dlPFC. This also took place in the precuneus, insula, ventromedial hypothalamus (VMH), dorsomedial hypothalamus (DMH) and RVLM (James et al. 2013; Macefield and Henderson 2019); connectivity analysis confirmed that each of these areas contributed to the generation of spontaneous bursts of MSNA (James et al. 2013; Macefield and Henderson 2019). Moreover, bursts of MSNA were temporally coupled to decreases in signal intensity in the NTS and CVLM, which fits with the basic medullary circuitry of the arterial baroreflex (Macefield and Henderson 2010).

Due to the dlPFC being one of the areas shown to contribute to the generation of MSNA, and it being a cortical area which is easily targeted for non-invasive electrical stimulation, recent discoveries within our laboratory showed that transcranial alternating current stimulation (tACS) of the dlPFC is, indeed, capable of producing marked modulation of sympathetic outflow (Sesa-Ashton et al. 2022; Wong et al. 2023). Moreover, in delivering concurrent sinusoidal electrical stimulation to the dlPFC and to the vestibular apparatuses, we have also recently found that sGVS-induced perceptions of sway and nausea are almost completely abolished by the stimulation of the dlPFC. We suggested that this reflects dlPFC-mediated top-down control of vestibular inputs (McCarthy et al. 2023a). As such, the present study aimed to expand upon this research with simultaneous stimulation of these sites alongside recordings of MSNA. Given that the central circuitry of the dlPFC has much in common with the vestibular system (McCarthy et al. 2023b), and that both structures are capable of modulating sympathetic activity, we proposed that simultaneous sGVS and tACS of the dlPFC would have an additive effect on MSNA. This would mean that when the two stimuli – of identical current and frequency (± 2 mA, 0.08 Hz) – were delivered concurrently, the magnitude of the cyclic modulation of MSNA would be higher than that produced by sGVS or stimulation of the dlPFC alone.

## Methods

### Participants and ethics

Studies were performed on eight male and three female subjects (age: 21–28 years, mean ± standard deviation (SD), 23.3 ± 2.6 years; height: 155–180 cm, 170.9 ± 8.9 cm; weight: 52–117 kg, 70.9 ± 18.1 kg), and included preliminary data previously acquired from six male and six female subjects (age: 22–32 years, 27.8 ± 4.0 years; height: 161–179 cm, 169.3 ± 5.7 cm; weight: 51–88 kg, 64.0 ± 12.7 kg) for a total sample size of 23. All participants provided informed written consent. The study was conducted with approval of the Human Research Ethics Committee of Western Sydney University (HREC approval H11010), endorsed by Governance of the Baker Heart and Diabetes Institute, and satisfied the Declaration of Helsinki. Prior to the commencement of the experiments, participants were instructed to refrain from smoking and the intake of caffeinated food and beverages, as well as to void their bladder. Each of these factors have been shown to have effects on sympathetic nerve activity (Fagius and Karhuvaara 1989; Grassi et al. 1994; Papadelis et al. 2003).

### Recording procedures

To begin the experiment, participants were seated in a semi-reclined position with their legs extended. The common peroneal nerve was initially located through manual palpation around the fibular head. Once found, the leg was supported by a casting cushion (Germa Protec, Sweden) in such a way as to allow for ease of access to the nerve and to minimise movements. A 35 mm adhesive hydrogel Ag/AgCl surface electrode (Covidien, Ireland) was then placed close to the knee to act as an anode for a search probe utilising short (0.2 ms) pulses of electrical current at 1 Hz delivered from an isolated stimulator (Stimulus Isolator, ADInstruments, Australia) to find the area of the nerve conveying the greatest sensitivity. This served as the site for percutaneous insertion of an insulated, sterilised tungsten microelectrode with a tip diameter of 200 μm (FHC, USA) into the right (*n* = 21) or left (*n* = 2) common peroneal nerve via which MSNA was recorded. A similar subcutaneous microelectrode (with 1 mm of insulation removed) was set approximately 1 cm away to act as a reference electrode. The recording microelectrode was then manually guided into a muscle fascicle using progressively weaker electrical stimuli (1.0 to 0.01 mA, 0.2 ms, 1 Hz) as it approached. Muscle twitches evoked with a current of ≤ 0.02 mA indicated that an appropriate fascicle had been entered. Neural activity was then amplified (gain 2 × 10^4^, bandpass 0.3 to 5.0 kHz) using a low-noise, electrically isolated headstage (NeuroAmpEX, ADInstruments, Australia). Real-time data were generated and connected to an audio system in order to hear bursts of MSNA, aiding in its identification. Sympathetic activity was tested for muscle properties with muscle stretch and a maximal inspiratory capacity apnoea. Evoked responses during either task indicated MSNA. Additionally, three 35 mm adhesive hydrogel Ag/AgCl surface electrodes (Covidien, Ireland) were placed on the torsos of participants for the purpose of acquiring recordings of the electrocardiogram (ECG) and sampled at 2 kHz. All physiological activity was visualised, recorded and stored using a computer-based data acquisition system (LabChart 7 for Macintosh, PowerLab 16 S; ADInstruments, Australia).

### Stimulation procedures

Sinusoidal, binaural stimulation (± 2 mA) at a frequency of 0.08 Hz was generated by stimulus isolators (Linear Stimulus isolator, World Precision Instruments, USA; Stimulus Isolator, ADInstruments, Australia) for 100 cycles of stimulation and applied to: (i) the dlPFC through tACS via 35 mm adhesive hydrogel Ag/AgCl surface electrodes (Covidien, Ireland) placed at EEG site F4 (as described by the international 10–20 system) and referenced to the nasion; (ii) the vestibular apparatuses through sGVS via matched Ag/AgCl surface electrodes placed on the mastoid processes; and (iii) both the dlPFC and vestibular apparatuses at the same time, with currents delivered in-phase with one another. Skin conductance electrode paste (Signa Cream, Parker Laboratories, USA) was used to improve contact between electrodes and the skin surface and a headband further ensured proper electrode correspondence. After a 10-minute baseline recording of MSNA, one of the three stimulation procedures would be implemented at random and would last for ~ 21-minutes. Each period of stimulation was separated by four minutes of rest.

### Data analysis

Analysis was performed on raw, negative-going MSNA spikes, ensuring that the data is not contaminated by the spikes produced by positive-going myelinated axons (Macefield and James 2016). Acquired negative-going spikes, R-waves of the ECG and peaks of the sinusoidally-delivered stimulation were detected using window discriminator software (Spike Histogram for Macintosh, v2.5.1, ADInstruments, Australia). Each of the data sets were used to generate autocorrelation and cross-correlation histograms using the same software. This enabled visualisation of patterns in each data set, which could then be compared using the autocorrelation histogram. Cross-correlation histograms (with time bins of 50 ms) were used to determine the influence of one variable upon another, namely: the influence of each of the three stimuli (sGVS, tACS and combined stimulation) on MSNA. Histogram data were exported as text to a statistical and graphical analysis program (Prism 8 for Windows, v8.4.3, GraphPad Software, USA). A smoothed polynomial was applied to each of the cross-correlation MSNA vs. stimulation graphs, eliminating cardiac related peaks. This allowed data to be more accurately analysed and peaks of MSNA to be more easily observed. The smoothed data were then used to calculate the modulation index of MSNA with respect to each stimulation method. The number of spikes in the 50 ms bin corresponding to the primary peak of modulation was compared to the number of spikes in the 50 ms bin corresponding to the trough of modulation according to the following formula: Modulation Index (%) = [(Peak-Trough)/Peak] x 100.

The latency and duration of expression of primary and secondary peaks of MSNA were also calculated using the smoothed polynomial data sets; as were the relationships between peaks and the phase of the sinusoid (i.e. whether or not the primary/secondary peak matched closely with the positive/negative phase of the sinusoid). Additionally, bursts of MSNA (obtained from the root mean square (RMS) of the nerve recording) and heart rate (obtained from the R-waves of the ECG) were gathered in blocks of three minutes before, at the start, and at the end of each stimulation procedure. These data were presented as ‘activity per minute’. All data were tested for normality using the D’Agostino and Pearson normality tests. In order to determine if there were significant differences (*P* < 0.05) in each of the measured parameters, an ordinary one-way analysis of variance (ANOVA) with a Tukey’s multiple comparisons test was used on unpaired data which passed normality, while repeated measures (RM) one-way ANOVA with a Holm-Šidák’s multiple comparisons test was used on paired data which passed normality. Furthermore, unpaired and paired data which failed to pass normality underwent Kruskal-Wallis tests and Friedman tests (both with a Dunn’s multiple comparisons test), respectively. A Wilcoxon t test was also used to compare paired modulation indices between sGVS and tACS of the dlPFC.

## Results

A total of 11 experiments were conducted, producing 11 sets of tACS of the dlPFC and combined tACS and sGVS data. Due to technical issues, there was a failure to record adequate sGVS data from two participants, therefore resulting in only nine sGVS data sets and, hence, nine data sets in which all three stimulation protocols were delivered. However, once unpublished data were included from 12 prior experiments, a total of 17 sGVS, 22 tACS and 11 combined stimulation data sets of MSNA were analysed from 22 participants. One participant was part of both the older (no combined stimulation procedure) and current experimental groups, and therefore, the total data set is *n* = 23. For this recurring participant, a period of eight months elapsed between experiments, which meant there was no possibility of any lasting stimulation effects skewing the data. In total, nine participants underwent all three stimulation protocols; seven participants underwent only sGVS and tACS of the dlPFC; two participants underwent only tACS of the dlPFC and combined sGVS and tACS; one participant underwent only sGVS; and four participants underwent only tACS of the dlPFC.

**Fig. 1:**
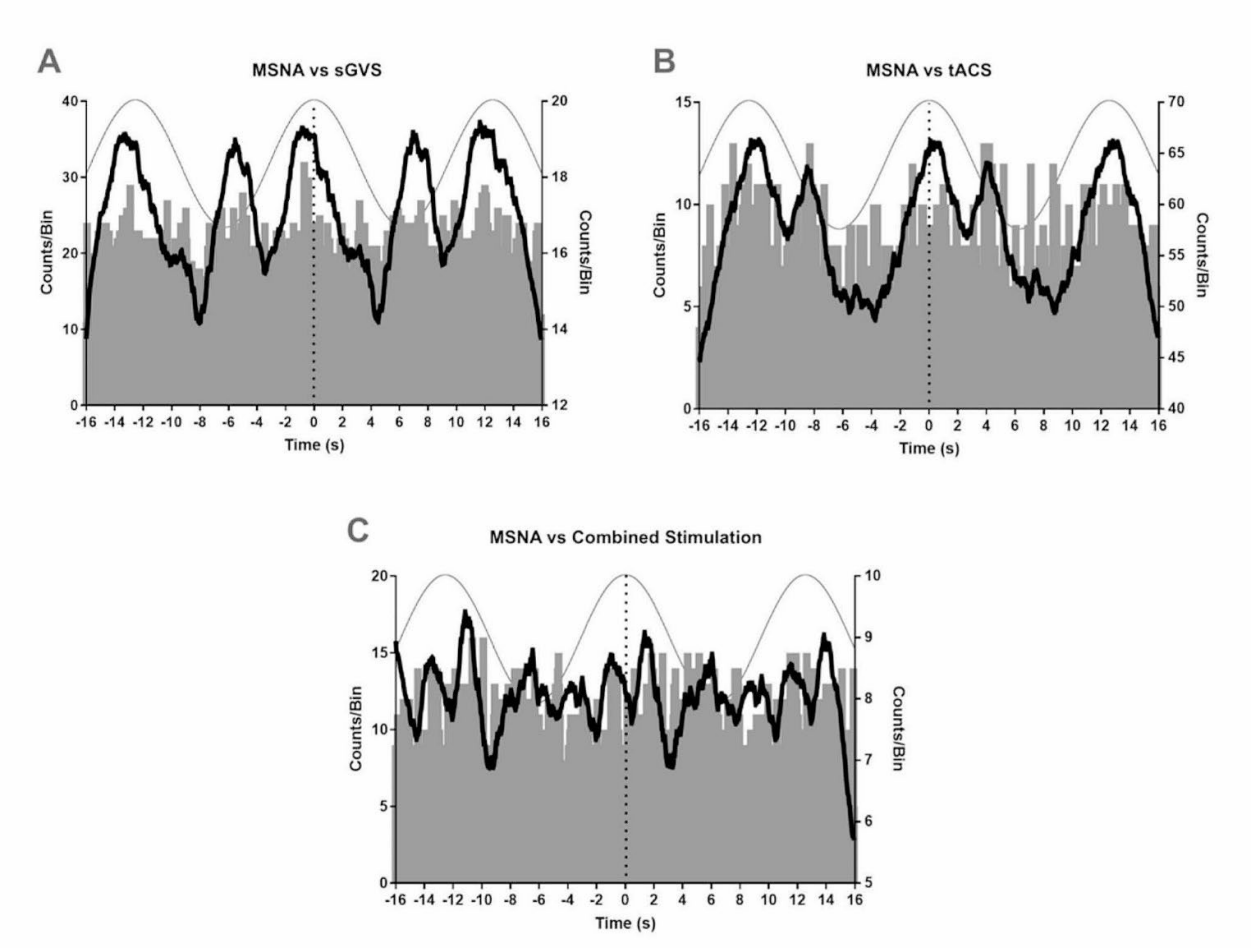
Cross-correlation histogram data obtained from a single female participant. (**A**) displays muscle sympathetic nerve activity (MSNA) data generated during sinusoidal galvanic vestibular stimulation (sGVS), (**B**) displays MSNA data obtained from transcranial alternating current stimulation (tACS) of the dorsolateral prefrontal cortex (dlPFC) and (**C**) displays MSNA data from combined stimulation measures. The black line overlayed on each graph denotes the smoothed polynomial and is set to the right Y-axis, while the left Y-axis represents raw data in 50 ms time bins. Data across a recording period were pooled to generate average activity at time zero, with average bursts prior and subsequent to this time point occurring during the negative and positive time periods, respectively

**Fig. 2:**
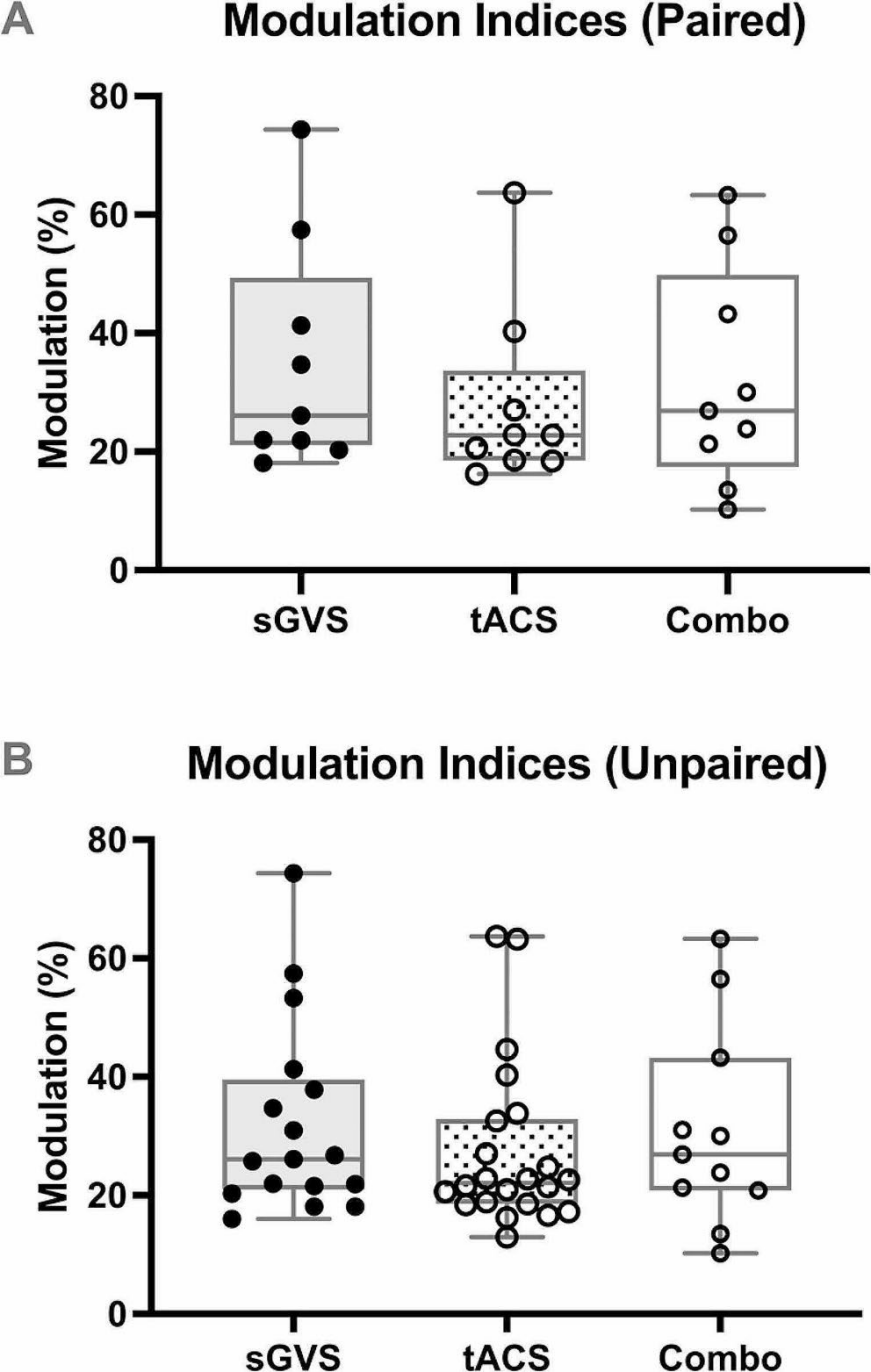
Modulation indices of primary peaks during each stimulation procedure. (**A**) shows the modulation of MSNA, as a percentage, specifically in the nine participants who underwent all three stimuli (Friedman test, *P* = 0.3977). P values for the paired data comparisons are as follows: sGVS vs. tACS, *P* = 0.4719; sGVS vs. Combo, *P* > 0.9999; tACS vs. Combo, *P* > 0.9999. (**B**) shows percentage of modulation of MSNA from every participant (*n* = 23), regardless of whether or not they underwent all three stimulation protocols (*n* = 17 for sGVS; *n* = 22 for tACS of the dlPFC; *n* = 11 for combined stimulation) (Kruskal-Wallis test, *P* = 0.5287). P values for the unpaired data comparisons are as follows: sGVS vs. tACS, *P* = 0.8552; sGVS vs. Combo, *P* > 0.9999; tACS vs. Combo, *P* > 0.9999. ‘Combo’ refers to combined sGVS and tACS stimulation. The box plots indicate the median, 25% and 75% confidence intervals. Error bars denote data points outside the interquartile range (IQR). No statistically significance (*P* > 0.05) differences were found between each group in either data set

Raw, negative-going MSNA spikes were analysed via cross-correlation histograms (Fig. 1). The smoothed polynomials of the 17 sGVS, 22 tACS and 11 combined stimulation data sets revealed a mean (± SD) modulation index of 32.2 ± 16.2%, 27.4 ± 14.1% and 31.0 ± 16.9% for each stimulation, respectively (Fig. 2). No statistically significant (*P* > 0.05) differences between the modulation indices of each of these groups were found (Kruskal-Wallis test, *P* = 0.5287). T test analysis (*P* = 0.2744) of the 16 paired sGVS (mean = 32.5 ± 16.7%) and tACS (mean = 30.8 ± 15.1%) modulation indices similarly led to a lack of significant differences (*P* > 0.05). Analysis (Friedman test, *P* = 0.3977) conducted on the modulation indices of the nine participants who underwent all three stimuli (comprising the paired data; mean values: sGVS = 35.2 ± 19.4%; tACS = 27.8 ± 15.2%; combined stimulation = 32.1 ± 18.5%) likewise displayed no significant differences (*P* > 0.05) across the protocols (Fig. 2).

**Fig. 3:**
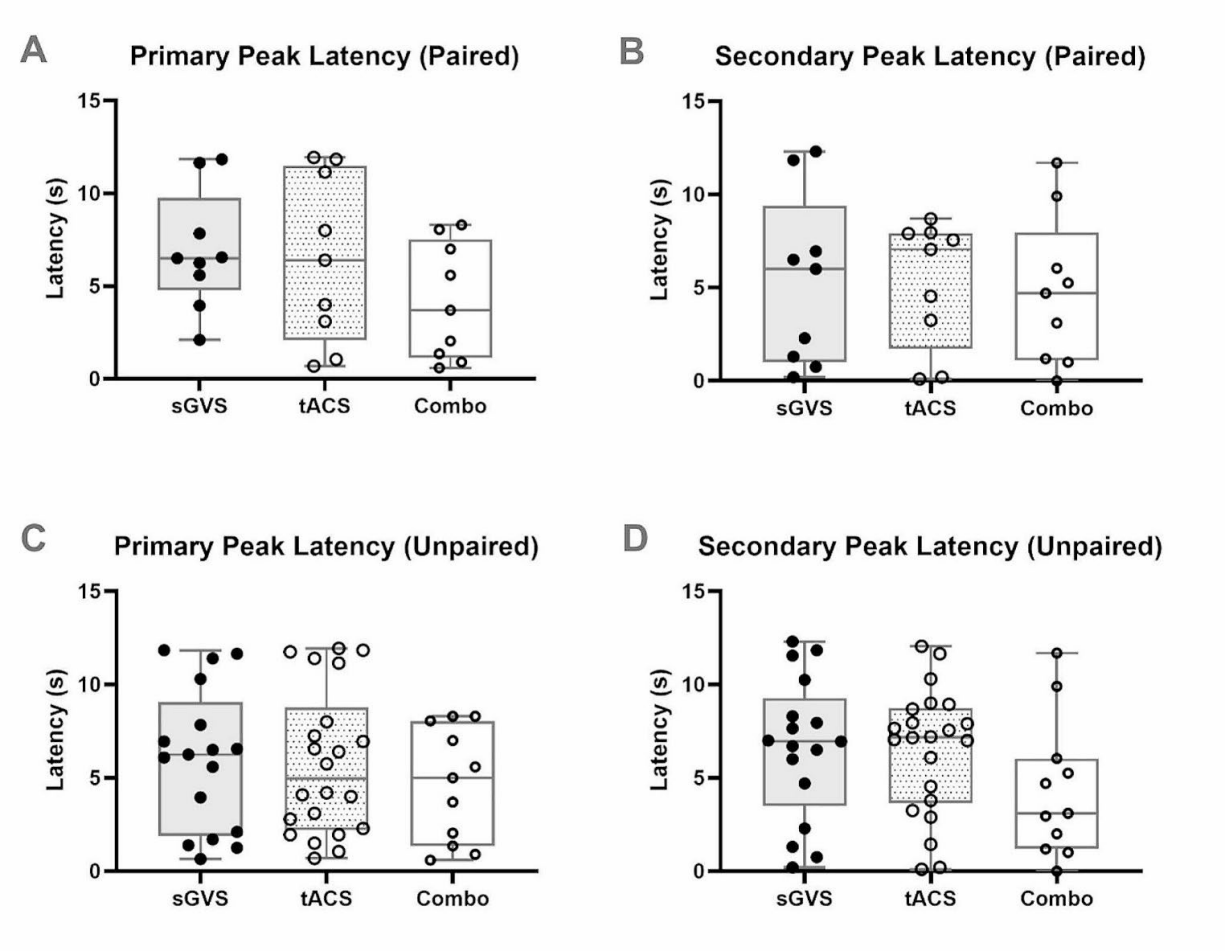
Latency of MSNA peak expression. (**A**) denotes the paired data (*n* = 9) for the time taken (seconds) for a primary peak of MSNA to appear during each stimulation procedure, with reference to cross-correlation histograms (RM one-way ANOVA, *P* = 0.2992). P values for the comparisons between stimulations are as follows: sGVS vs. tACS, *P* = 0.9752; sGVS vs. Combo, *P* = 0.2251; tACS vs. Combo, *P* = 0.4429. (**B**) presents paired data (*n* = 9) of the latency for secondary peaks (RM one-way ANOVA, *P* = 0.9305). P values for the comparisons between stimulations are as follows: sGVS vs. tACS, *P* = 0.9990; sGVS vs. Combo, *P* = 0.9556; tACS vs. Combo, *P* = 0.9497. Unpaired (*n* = 17 for sGVS; *n* = 22 for tACS of the dlPFC; *n* = 11 for combo) primary peak latency is depicted in (**C**) (ordinary one-way ANOVA, *P* = 0.6037), alongside unpaired secondary peak latency in (**D**) (ordinary one-way ANOVA, *P* = 0.2146). P values for the unpaired primary peak comparisons are as follows: sGVS vs. tACS, *P* = 0.9766; sGVS vs. Combo, *P* = 0.5994; tACS vs. Combo, *P* = 0.6834. P values for the unpaired secondary peak comparisons are as follows: sGVS vs. tACS, *P* = 0.9933; sGVS vs. Combo, *P* = 0.2463; tACS vs. Combo, *P* = 0.2542. ‘Combo’ refers to combined sGVS and tACS stimulation. The box plots indicate the median, 25% and 75% confidence intervals. Error bars denote data points outside the IQR. No significant (*P* > 0.05) differences were observed between each method of stimulation in both the paired and unpaired data sets

The smoothed polynomials were also used to analyse the latency of the expression of primary and secondary peaks of MSNA in participants who underwent the combined stimulation protocol (Fig. 3). Latency analysis (Primary peaks: RM one-way ANOVA, *P* = 0.2992; secondary peaks: RM one-way ANOVA, *P* = 0.9305) of the nine paired sGVS recordings showed a mean (± SD) latency of 6.9 ± 3.2 s for the expression of a primary peak, whilst the secondary peak was expressed at the mean latency of 5.4 ± 4.6 s. The nine corresponding dlPFC tACS recordings displayed a primary peak at a mean of 6.5 ± 4.5 s and a secondary peak at a mean of 5.3 ± 3.4 s. Primary peaks at 4.2 ± 3.1 s and secondary peaks at 4.8 ± 4.0 s were observed in the nine paired recordings from the combined stimulation. Though the average values are as stated, there was a great deal of variance in these results. The secondary peaks of sGVS, for example, had a range expressed over 12.1 s. Statistical analysis revealed no significant differences (*P* > 0.05) between any of the mean latencies. The same results were found in analysis of all the primary (ordinary one-way ANOVA, *P* = 0.6037) and secondary peaks in the unpaired data (ordinary one-way ANOVA, *P* = 0.2146) of sGVS (*n* = 17) (means = 6.0 ± 3.8 s; 6.6 ± 3.8 s), tACS (*n* = 22) (means = 5.8 ± 3.9 s; 6.5 ± 3.4 s), and combined stimulation (*n* = 11) (means = 4.6 ± 3.1; 4.4 ± 3.7) (Fig. 3).

**Fig. 4:**
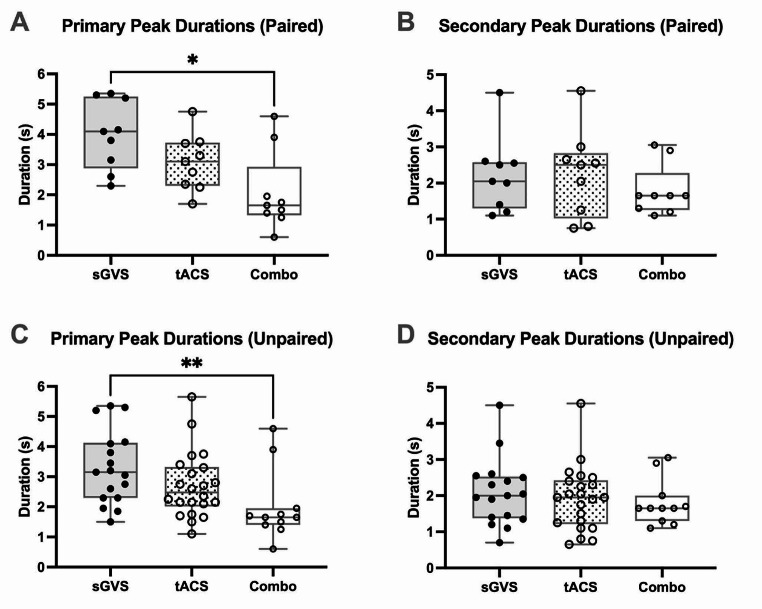
Durations of expression of MSNA peaks. (**A**) shows paired data (*n* = 9) of the duration, in seconds, of primary peaks of MSNA in each of the three stimuli (RM one-way ANOVA, *P* = 0.0139). P values for the comparisons between stimulations are as follows: sGVS vs. tACS, *P* = 0.0797; sGVS vs. Combo, *P* = 0.0318; tACS vs. Combo, *P* = 0.2384. (**B**) presents the duration of the secondary peaks from the same data sets (*n* = 9) (Friedman test, *P* = 0.9712). P values for the comparisons between stimulations are as follows: sGVS vs. tACS, *P* > 0.9999; sGVS vs. Combo, *P* > 0.9999; tACS vs. Combo, *P* > 0.9999. Peak duration is calculated as the time between the apex and trough of the peak of MSNA modulation, generated via cross-correlation histograms. A significant difference was found between sGVS and combined stimulation primary peaks in this paired data set (*P = 0.0318). (**C**) and (**D**) display unpaired primary (Kruskal-Wallis test, *P* = 0.0067) and secondary (Kruskal-Wallis test, *P* = 0.6785) peak duration, respectively, across all acquired data from the 23 experiments. P values for the unpaired primary peak comparisons are as follows: sGVS vs. tACS, *P* = 0.4363; sGVS vs. Combo, *P* = 0.0046; tACS vs. Combo, *P* = 0.1228. P values for the unpaired secondary peak comparisons are as follows: sGVS vs. tACS, *P* > 0.9999; sGVS vs. Combo, *P* > 0.9999; tACS vs. Combo, *P* > 0.9999. A significant difference was found between sGVS and combined stimulation primary peaks in this unpaired data set (**P = 0.0046). ‘Combo’ refers to combined sGVS and tACS stimulation. The box plots indicate the median, 25% and 75% confidence intervals. Error bars denote data points outside the IQR

The duration of the peaks (from the apex to the trough) was determined from the smoothed polynomials in the nine participants who underwent all three stimuli as well (Fig. 4). The mean (± SD) duration of the primary and secondary peaks during sGVS were 4.0 ± 1.2 s and 2.2 ± 1.0 s, respectively. The dlPFC tACS primary peaks had a mean duration of 3.1 ± 0.9 s, while the secondary peaks had a mean duration of 2.2 ± 1.2 s. Combined stimulation resulted in primary peaks occurring for a mean of 2.1 ± 1.3 s and secondary peaks for 1.8 ± 0.7 s. Multiple comparisons analysis (primary peaks: RM one-way ANOVA, *P* = 0.0139; secondary peaks: Friedman test, *P* = 0.9712) of the paired data revealed a statistically significant (*P* = 0.0318) difference between sGVS and combined stimulation primary peaks. This was also seen in sGVS primary peaks vs. combined stimulation secondary peaks (*P* = 0.0158), however, given that this is a comparison between primary and secondary peaks, this difference is likely irrelevant. Primary (Kruskal-Wallis test, *P* = 0.0067) and secondary (Kruskal-Wallis test, *P* = 0.6785) peaks of sGVS (*n* = 17) (means = 3.3 ± 1.2 s; 2.1 ± 0.9 s), tACS (*n* = 22) (means = 2.7 ± 1.1 s; 1.9 ± 0.9 s), and combined stimulation (*n* = 11) (means = 2.0 ± 1.2 s; 1.8 ± 0.6 s) likewise revealed a significant difference (*P* = 0.0046) in the unpaired data set: multiple comparisons showed that the duration of combined stimulation primary peaks was significantly shorter than that of the sGVS primary peaks (Fig. 4), while there were no significant differences (*P* > 0.05) between primary peaks in dlPFC tACS and combined stimulation.

**Fig. 5:**
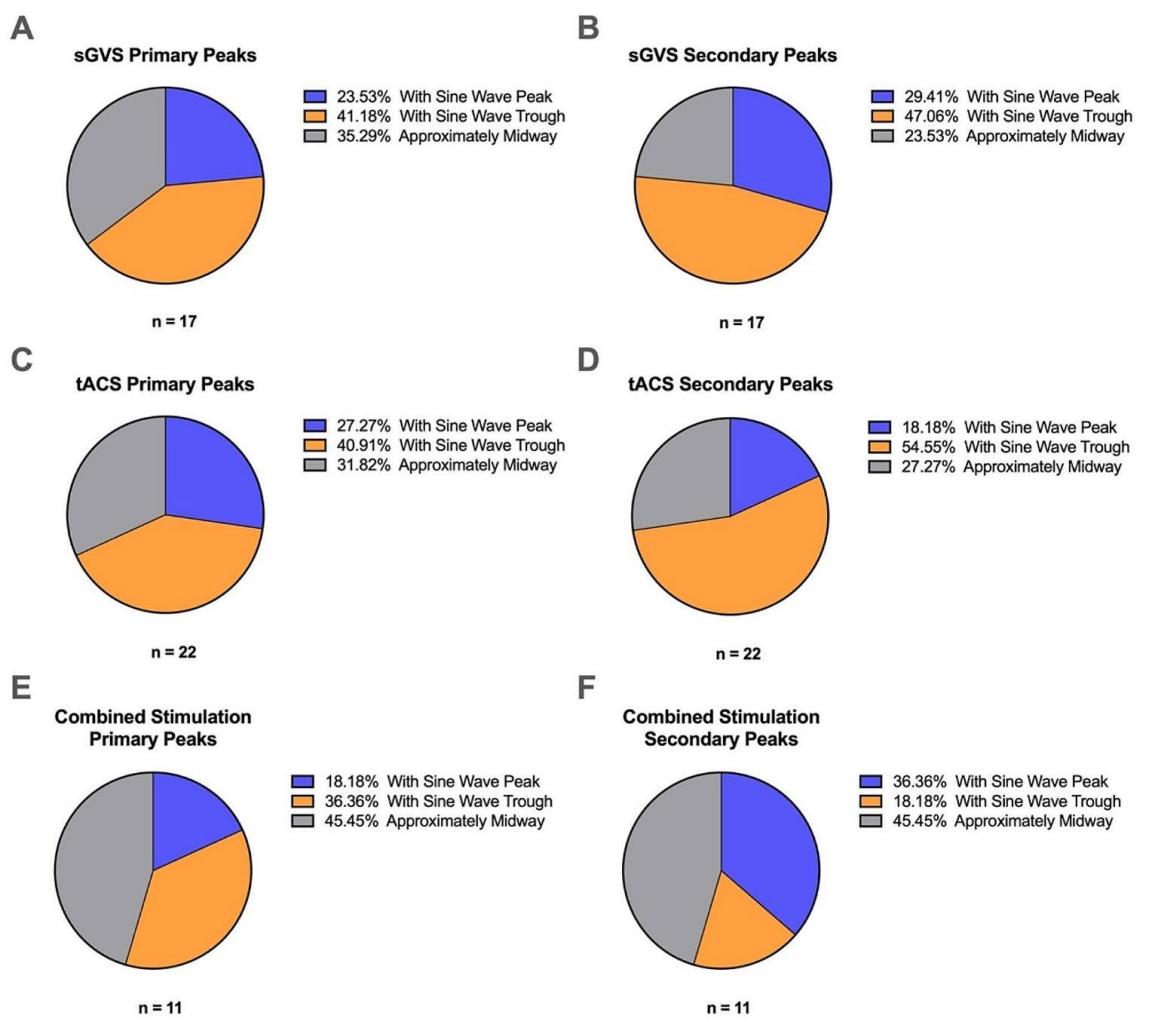
Relationships between MSNA peaks and sine waves. The charts in (**A**) and (**B**) display the occurrence of primary and secondary peaks of MSNA, respectively, with regards to the phase of the sinusoidal stimulation during sGVS (*n* = 17). (**C**) shows primary and (**D**) shows secondary peaks of MSNA during tACS of the dlPFC (*n* = 22). Primary and secondary peaks of MSNA during combined stimulation (*n* = 11) are seen in charts (**E**) and (**F**). MSNA peaks were defined as occurring during the peak of the sine wave, the trough of the sine wave or approximately midway between the peak and trough, as seen via cross-correlation histograms. Analysis (paired data: Friedman test, *P* = 0.7212; unpaired data: Kruskal-Wallis test, *P* = 0.5709) of the patterns of occurrence revealed no statistical significance (*P* > 0.05) between each group

Additionally, the occurrence of the primary and secondary peaks during each stimulation procedure (all 17 sGVS, 22 tACS and 11 combined stimulations) was analysed (Kruskal-Wallis test, *P* = 0.5709) with respect to the phase of the sinusoidal stimulus (Fig. 5). Peaks of MSNA were grouped in one of three ways: with the sine peak, with the sine trough, and approximately midway between the sine peak and trough. Statistical analysis revealed no significant differences (*P* > 0.05) in the pattern of expression of MSNA between each group. Another test run on the nine sets of paired data (Friedman test, *P* = 0.7212) similarly returned results with no significant differences (*P* > 0.05).

**Fig. 6:**
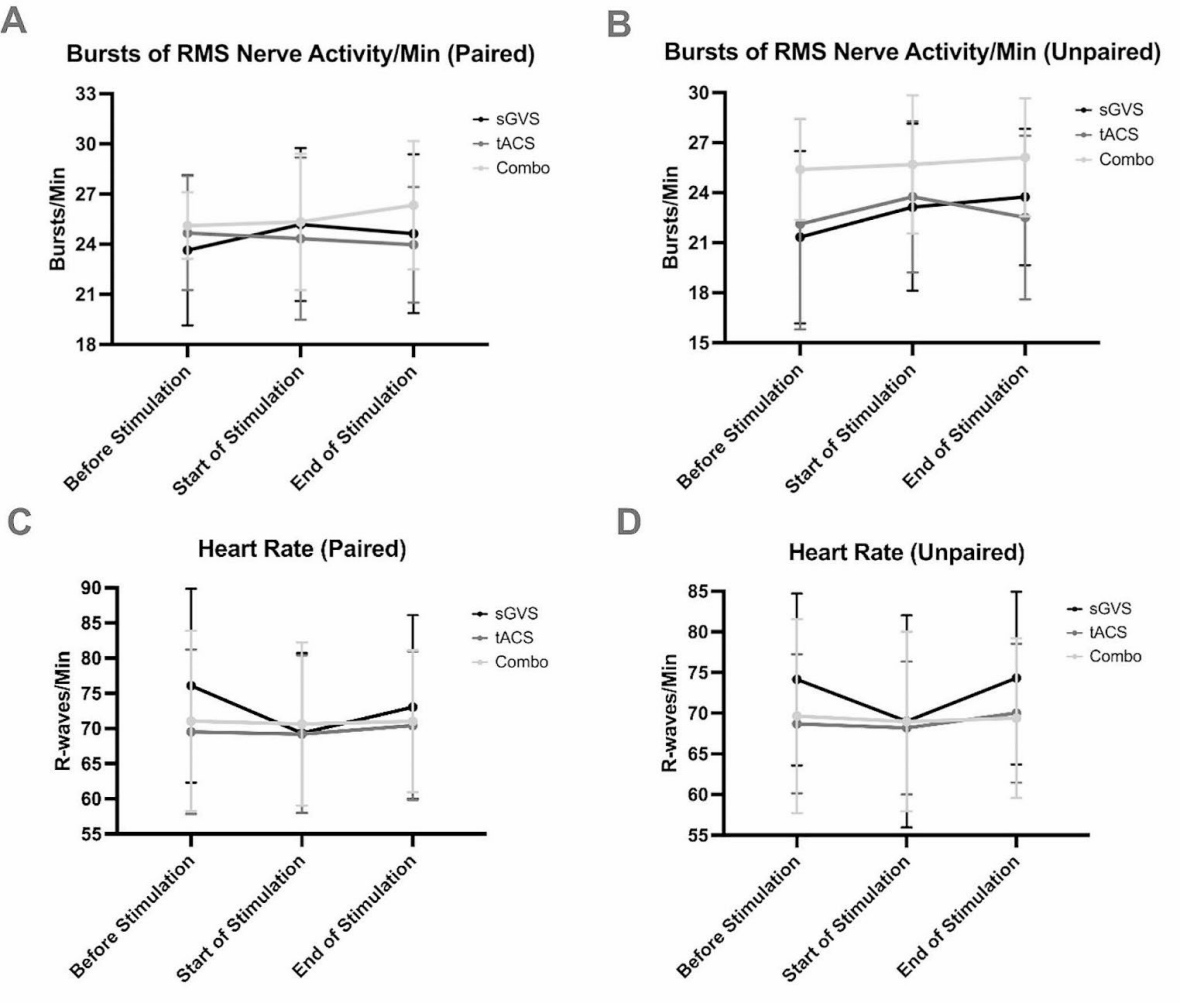
MSNA burst frequency and heart rate. Each graph represents three-minute time periods before, at the start, and at the end of each sGVS, tACS of the dlPFC, and combined stimulation protocol. (**A**) displays MSNA bursts per minute (calculated from the root mean square (RMS) of the raw nerve activity) in the paired (*n* = 9) data set (RM one-way ANOVA, *P* = 0.6080), while (**B**) represents unpaired sGVS (*n* = 17), tACS (*n* = 22), and combined stimulation (*n* = 11) data (ordinary one-way ANOVA, *P* = 0.1022). Paired (RM one-way ANOVA, *P* = 0.0895) and unpaired (ordinary one-way ANOVA, *P* = 0.5041) heart rate data are shown in (**C**) and (**D**), respectively. No significant (*P* > 0.05) differences were found at any time across or between any of the stimulation protocols. Data are presented as mean values with error bars denoting standard deviation (SD)

Furthermore, bursts of MSNA (as measured from the RMS of the raw nerve signal) were evaluated in three-minute time periods immediately before, at the very beginning, and at the very end of each stimulation procedure. This enabled MSNA to be calculated as bursts per minute. The mean (± SD) MSNA data in both the paired (RM one-way ANOVA, *P* = 0.6080) and unpaired (ordinary one-way ANOVA, *P* = 0.1022) data sets are displayed in Table 1. No significant differences (*P* > 0.05) in MSNA levels across each time period were found (Fig. 6).

**Table 1:** Mean MSNA bursts per minute. The paired (RM one-way ANOVA, *P* = 0.6080; *n* = 9) and unpaired (ordinary one-way ANOVA, *P* = 0.1022; *n* = 17 for sinusoidal galvanic vestibular stimulation (sGVS); *n* = 22 for transcranial alternating current stimulation (tACS) of the dorsolateral prefrontal cortex (dlPFC); *n* = 11 for combined stimulation) mean MSNA bursts per minute data (calculated from the root mean square (RMS) of the raw nerve activity) from three-minute time periods before, at the start, and at the end of each stimulation protocol is displayed. For both the paired and unpaired data, no statistically significant differences (*P* > 0.05) were found between any stimulation conditions. ‘Combo’ refers to combined sGVS and tACS stimulation. Data are presented as mean ± standard deviation (SD)

	sGVS		tACS		Combo	
	Paired	Unpaired	Paired	Unpaired	Paired	Unpaired
Before Stimulation	23.6 ± 4.5	21.3 ± 5.2	24.7 ± 3.4	22.1 ± 6.3	25.1 ± 2.0	25.4 ± 3.0
Start of Stimulation	25.2 ± 4.6	23.1 ± 5.0	24.3 ± 4.9	23.8 ± 4.5	25.3 ± 4.1	25.7 ± 4.1
End of Stimulation	24.6 ± 4.7	23.8 ± 4.1	24.0 ± 3.5	22.5 ± 4.9	26.3 ± 3.8	26.1 ± 3.5

The mean (± SD) heart rate in the nine individuals with paired data (RM one-way ANOVA, *P* = 0.0895) are presented in Table 2. Just as with the burst analysis of MSNA, R-waves of the ECG were determined during three-minute periods before, at the start, and at the end of each stimulation protocol. Multiple comparisons revealed no statistically significant (*P* > 0.05) differences. The unpaired heart rate data (similarly shown in Table 2), once analysed (ordinary one-way ANOVA, *P* = 0.5041), were also not significantly different (*P* > 0.05) in all of the multiple comparisons conducted (Fig. 6).

**Table 2:** Mean heart rate. The paired (RM one-way ANOVA, *P* = 0.0895; *n* = 9) and unpaired (ordinary one-way ANOVA, *P* = 0.5041; *n* = 17 for sGVS; *n* = 22 for tACS; *n* = 11 for combo) mean heart rate from three-minute periods before, at the start, and at the end of each stimulation protocol is displayed. No statistically significant differences (*P* > 0.05) were found between any points. ‘Combo’ refers to combined sGVS and tACS stimulation. Data are presented as mean ± SD

	sGVS		tACS		Combo	
	Paired	Unpaired	Paired	Unpaired	Paired	Unpaired
Before Stimulation	76.1 ± 13.8	74.2 ± 10.6	69.5 ± 11.7	68.7 ± 8.6	71.0 ± 12.8	69.6 ± 11.9
Start of Stimulation	69.4 ± 11.4	69.0 ± 13.0	69.2 ± 11.2	68.2 ± 8.2	70.6 ± 11.6	69.0 ± 11.0
End of Stimulation	73.1 ± 13.1	74.3 ± 10.6	70.4 ± 10.6	70.0 ± 8.5	71.0 ± 10.1	69.4 ± 9.8

## Discussion

The objective of the present study was to gain an understanding of dlPFC contributions to the regulation of vestibulosympathetic reflexes, as evidenced by the effects of tACS of the dlPFC on the vestibular modulation of MSNA produced by sGVS. It was recent research conducted in our laboratory which first discovered that the vestibular illusions of side-to-side movement – induced by sGVS – were abolished by concurrent tACS of the dlPFC (McCarthy et al. 2023a). The data presented in the current work are the first to investigate the effects of dlPFC stimulation on MSNA in combination with sGVS. It expands upon a wealth of prior research from our laboratory into the coupling of vestibulosympathetic reflexes to the cardiovascular system, as recorded through MSNA (Bent et al. 2006; Grewal et al. 2009; Hammam et al. 2011, 2014; Hammam and Macefield 2017; James and Macefield 2010; Macefield and James 2016) and dlPFC-mediated control of sympathetic activity (Sesa-Ashton et al. 2022; Wong et al. 2023).

### Vestibular and dlPFC interactions

It has been previously established in many studies that MSNA undergoes robust modulation during low-frequency sGVS (Grewal et al. 2009; Hammam et al. 2011; Macefield and James 2016), with frequencies below 0.2 Hz eliciting the expression of secondary peaks of modulation (Hammam et al. 2011). The current study has found, however, that tACS of the ipsilateral dlPFC (with regards to the microneurographic recording site) on its own at 0.08 Hz will produce equivalent changes to levels of MSNA as produced by sGVS at the same frequency. Indeed, this stimulation was even shown to elicit distinct secondary peaks of modulation as well. Comparisons of paired as well as unpaired modulation indices between sGVS and tACS yielded no significant differences. This lack of significance, alongside the appearance of secondary peaks, shows that the dlPFC-controlled modulation of MSNA is just as robust as the vestibular modulation. Importantly, the combined stimulation protocol expressed the same trend as seen in stimulation of the vestibular apparatuses and the dlPFC as well. Modulation of MSNA remained the same; the two systems did not have an additive influence on each other (contrary to our initial hypothesis), nor did they cancel each other out. The absence of an increased or decreased modulation index strongly implies a disturbance in the higher order processing of sympathetic outflow.

The results seem to suggest that the vestibular contributions to the regulation of blood flow are being blocked by the dlPFC during the combined stimulation protocol. This is in line with our previous findings that the dlPFC was inhibiting vestibular-induced nausea and perceptions of sway (McCarthy et al. 2023a). While that paper explores possible dlPFC influences on higher cortical regions, these new results, however, suggest that the central sympathetic connectome at the level of the brainstem may be playing a key role in this process. It would appear that the vestibular afferent projections from the caudal vestibular nuclei to the NTS, CVLM, and RVLM in particular, may be inhibited by dlPFC activity.

One possible explanation of this dlPFC inhibitory function lies in upregulation of the gamma-aminobutyric acid (GABA) inhibitory pathway centring around the CVLM and RVLM (Agarwal et al. 1989, 1990). Increasing GABA transmission can impair the ability of the vestibular system to generate MSNA. Due to the aforementioned lack of an additive influence on MSNA modulation during the combined stimulation protocol (with evidence that modulation does, indeed, still take place, but not according to the initially hypothesised additive effect), there is an implication that some form of partial sympathetic inhibition is taking place. Holstein et al. (2016) provide evidence that the vestibulosympathetic reflexes themselves are influenced by GABA transmission from the vestibular nuclei to the RVLM during sGVS. This is usually vastly outcompeted by the GABA transmission from the same vestibular nuclei to the CVLM, which reduces the CVLM inhibitory properties and allows for increased MSNA modulation. However, if the dlPFC can upregulate this sGVS-induced GABA to RVLM pathway, this may account for the results found in the present study. Through this, the dlPFC may be capable of increasing MSNA when stimulated on its own, and function to offset the vestibulosympathetic reflexes when stimulated in conjunction with the vestibular system – having no effect on upregulating vestibular-induced GABA to RVLM functions if the vestibular system is not concurrently engaged. Electrical stimulation of the dlPFC has, in fact, been demonstrated to increase GABA transmission in the past, albeit to the striatum rather than the brainstem (Bunai et al. 2021). Regardless, this provides evidence that the dlPFC is capable of mediating GABA transmission.

Moreover, the insular cortex (IC) may be playing an important role here. As we have previously discussed (McCarthy et al. 2023a), the insula has interconnections to both the vestibular system (Akbarian et al. 1993; Chen et al. 2010; Guldin and Grüsser 1998) and the dlPFC (Fu et al. 2021; Gao et al. 2021; Steward et al. 2016), which led us to believe that it may be a key structure in the abolition of vestibular-induced nausea and perceptions of sway. Additionally, in the fact that the insula has been well established to have cardiovascular control (Cechetto and Chen 1990; Chouchou et al. 2019), and in light of our newly acquired results, the possibility of its involvement in our stimulation protocol is further justified. In rats, the intermediate portion of the IC has been shown to have direct projections to the RVLM, while both the intermediate as well as the rostral regions have projections towards the CVLM (Marins et al. 2016). By injecting an excitatory neurotransmitter into the rostral region of the IC, Marins et al. (2016) noted a marked reduction in heart rate and renal sympathetic nerve activity via the CVLM. This sympathetic alteration was seen in the opposite manner with neurotransmitter injection into the intermediate IC; heart rate and renal sympathetic nerve activity increased by means of the RVLM. Here, as with our GABA upregulation hypothesis, we may find evidence as to why there were no additive influences on MSNA between the dlPFC and vestibular system during our combined stimulation protocol. If the dlPFC has an excitatory influence on the rostral IC (and, hence, the CVLM) in a similar fashion to that found in these rats, or an inhibitory influence on the intermediate IC (hence the RVLM), we may expect to see a suppression of the vestibulosympathetic responses. The dlPFC may be barring the vestibular sympathetic pathway in particular – not the generalised MSNA pathway through which the dlPFC is capable of increasing MSNA when stimulated on its own (Sesa-Ashton et al. 2022). An inhibitory response on the RVLM through the IC would be consistent with one of our last hypotheses that downregulation of insula activity could ‘gate’ vestibular signal propagation (Huang et al. 2021; McCarthy et al. 2023a).

The latency data of MSNA peak expression reinforces the notion of a blockade of vestibular signals. Much like the modulation indices data, there were no significant differences between stimulation protocols in the latency of MSNA primary and secondary peaks. This supports our conclusion that the dlPFC does not work with or against the vestibular system, per se, rather that it prevents vestibular signalling altogether. Had the combined stimulation data shown a greater correlation to the tACS data, and by proxy been significantly different to the sGVS data, this would be further supported.

Data on the duration of primary and secondary peaks of MSNA likewise showed a lack of significant differences between the stimulation paradigms, with an exception. Both the paired and unpaired data revealed a significant difference between sGVS and combined stimulation primary peaks. This means that the combined stimulation, at least in this aspect, does indeed more closely resemble the patterns of tACS rather than sGVS – it was not different to dlPFC stimulation, but was to sGVS. Granted, had the sGVS vs. tACS data also reached a statistically significant difference, this claim would be more well-supported. However, the P value of 0.0797 between these two stimulations shows that it was close to doing so. If the vestibular system had been a more dominant force driving the generation of MSNA during combined stimulation, we would expect to see longer peak durations that are more akin to those generated by sGVS.

Patterns of primary and secondary MSNA peak expression relative to the phase of the sinusoidal stimulus were also analysed to support the theory. MSNA peaks appeared to have a greater correlation with the sine trough during sGVS and tACS, while combined stimulation peaks appeared to correlate with the middle of the sine wave. As such, in this analysis parameter, there may be some evidence of the vestibular system carrying out a modulatory effect during combined stimulation after all, albeit a severely diminished one. This coincides with our previous study (McCarthy et al. 2023a) in which some participants still reported mild vestibular sensations during the combined stimulation. It would be expected that MSNA peak patterning, too, would have a higher correlation with the sine trough if vestibular influences were completely abolished during combined stimulation. As this is not the case, there remains a possibility that the vestibular system is at conflict with the dlPFC, resulting in the impairment of MSNA peak timing relative to the sinusoidal stimulus. These findings, though, are somewhat dissimilar to another study which concluded that primary peaks correlated with the sine peak and secondary peaks correlated with the sine trough during sGVS (Hammam et al. 2011). There is the possibility, however, that this may be attributed to a grouping system the study utilised that only accounted for the peak and trough of the sinusoid, with no midway parameter. While the pattern results obtained in our experiments did not reach statistical significance, the absence of significance does, however, further prove that tACS of the dlPFC is equivalent to sGVS in the regulation of MSNA in almost every aspect. Furthermore, the absence of significance also provides more evidence to support the idea that the modulation produced from combined stimulation could be solely derived from the dlPFC, without any vestibular influences. With this being said, the process of determining which category each peak fell into was subjective. Categorisation was determined by visuals alone, and a few peaks proved to be ambiguous as to which group they should be placed in.

It is also worth elaborating upon the fact that our combined stimulation protocol used two sets of stimulations delivered with the sinusoidal currents in-phase. This was the same method as used previously (McCarthy et al. 2023a) to show dlPFC inhibition of sGVS-induced vestibular illusions, however it may prove interesting to conduct further study into the effects of a phase lag stimulation – especially with regards to MSNA. As Alekseichuk et al. (2019) reported, in-phase currents applied to two brain regions will typically increase coordination, with out-of-phase currents producing opposite effects. Exactly how a current with phase lag applied to the dlPFC may affect the vestibulosympathetic reflexes, and the secondary peaks of MSNA in particular, is grounds for consideration. Should the brain regions begin to act asynchronously, secondary peaks of activity may disappear in favour of primary peaks from each brain region in an interleaved pattern (e.g. a dlPFC-generated primary peak followed by a vestibular-generated primary peak, instead of a secondary peak). This, of course, may also depend on the degree of phase lag and may, indeed, influence vestibular perceptions of sway as well (McCarthy et al. 2023a).

Moreover, in seeking to further determine the similarities of the dlPFC and vestibular system in the generation of MSNA, MSNA bursts (obtained from the RMS of the nerve signal) and heart rate (obtained from the ECG) were analysed, as it is known that the vestibular-mediated control of MSNA is linked to the cardiac cycle (Hammam and Macefield 2017). The three-minute periods before, at the start, and at the end of each stimulation protocol were analysed to generate the number of bursts of MSNA for each period. In a few cases, due to technical issues, entire three-minute periods were unavailable, but calculations were adjusted to account for this. There were no statistically significant changes in MSNA burst frequency or in heart rate across each of the time periods for each stimulation paradigm, supplementing the idea of the equality of the dlPFC to the vestibular system in MSNA modulation. While it may not be statistically significant, a look into the patterns of correlation between bursts of MSNA and heart rate reveals some possible insights. In the paired data set, there appears to be an inverse relationship between MSNA and heart rate during sGVS, which is similar to the findings of previous studies (Charkoudian et al. 2005; Hart et al. 2009) noting that, in men, an inverse relationship exists between MSNA and cardiac output (which, itself, is dependent on heart rate and stroke volume). During tACS of the dlPFC, both the paired and unpaired data sets seem to suggest a less pronounced inverse relationship, but one that exists nonetheless. However, this relationship appears to be even less pronounced during combined stimulation. Here, as with the MSNA peak relationship data, we may find evidence to support the idea of the vestibular system having a minor influence in preventing the dlPFC from having complete control during combined stimulation. Though it might be expected that vestibular influences would increase the inverse relationship, the vestibular system may, in fact, be suppressing the dlPFC to a minimal degree.

### Limitations

Just as with our previous paper on vestibular perception interference via the dlPFC (McCarthy et al. 2023a), a possible confounding variable in this work is found in the ventromedial prefrontal cortex (vmPFC). In stimulating the vestibular apparatuses via sGVS, our study circumvents issues of older vestibular stimulation methods known to induce off-target effects such as fluid shifts in the body (Cui et al. 2001; Kaufmann et al. 2002) and reflexive responses from muscles of the neck (Bolton and Ray 2000). These are capable of interfering with vestibular and MSNA recordings. Yet, in stimulating the dlPFC in a similar way via tACS referenced towards the nasion, current travelled through the vmPFC, possibly activating it in the process. This is important with regards to a recent study conducted in our laboratory which showed the vmPFC to be capable of MSNA modulation to a similar extent as the dlPFC (Braun et al. 2024). Whether or not the vmPFC was activated and subsequently played a role in the observed interactions during the present study remain unknown – rendering it something worth bearing in mind.

Another point to consider is the diversity of the sample used in this project. The previously acquired data used to supplement the data set had an even spread of six male and six female subjects, but the newly acquired combined stimulation data was gathered from eight male and three female subjects. Incomplete data were collected from two of the female participants, which resulted in paired data being generated from a sample of eight males and one female. Alongside being a skewed representation of the general population, it has been previously established that arterial pressure linked to MSNA is sex-dependent (Hart et al. 2009). Men generally present with a higher mean resting MSNA and an inverse relationship between MSNA and cardiac output not seen in women (Hart et al. 2009). For these reasons, it is conceivable that the paired data on heart rate and MSNA reflect reduced changes in heart rate and higher changes in MSNA across the stimulation paradigms when compared to the population mean.

Finally, we did not include a placebo stimulation protocol, but refer the reader to our previous work (Sesa-Ashton et al. 2022) in which dlPFC modulation of MSNA was shown to be site-specific – stimulation of the motor cortex (referenced to the vertex) produced no modulation.

## Conclusions

Our project has established for the first time a link between the vestibular system and the dlPFC in the control of MSNA. Levels of modulation of MSNA do not appear to be different during sGVS, tACS of the dlPFC, or a combined stimulation protocol, contrary to our initial thoughts. Similarly, there are no apparent differences in the latency of primary and secondary peak expression, as well as in their expression relative to the sinusoidal stimulus during all stimulation paradigms. MSNA burst incidence and heart rate likewise failed to show significant changes between the three stimulation protocols. The results did show, however, that the duration of primary peaks differed significantly between sGVS and combined stimulation, supporting our interpretation that the dlPFC is the more dominant driver of MSNA during concurrent sGVS and tACS. These results are in line with the results of our previous study, which demonstrated inhibition of sGVS-induced perceptions of sway and nausea – intrinsic characteristics of sGVS – by the dlPFC (McCarthy et al. 2023a). Through a lack of significant differences in the present study, combined stimulation closely resembled the patterns of tACS of the dlPFC across all the measured parameters. Due to these reasons, we believe that the processing of vestibular information regarding sympathetic activity is being interrupted at the level of the brainstem, or perhaps through the insula. With this being said, the patterns of MSNA peaks relative to the sinusoidal stimulus and the inverse relationships seen between MSNA and heart rate suggest that the vestibular system may still have a minimal contribution to MSNA during combined stimulation.

## Data Availability

The data are available upon reasonable request.
